# ANO7 expression in the prostate modulates mitochondrial function and lipid metabolism

**DOI:** 10.1186/s12964-025-02081-7

**Published:** 2025-02-08

**Authors:** Christoffer Löf, Nasrin Sultana, Neha Goel, Samuel Heron, Gudrun Wahlström, Andrew House, Minna Holopainen, Reijo Käkelä, Johanna Schleutker

**Affiliations:** 1 Institute of Biomedicine, University of Turku, Kiinamyllynkatu 10, Turku, 20520 Finland; 2 FICAN West Cancer Center, University of Turku and Turku University Hospital, Kiinamyllynkatu 10, Turku, 20520 Finland; 3 Helsinki University Lipidomics Unit (HiLIPID), Helsinki Institute of Life Science (HiLIFE) and Biocenter Finland, University of Helsinki, Viikinkaari 1, P.O. Box 65, Helsinki, 00014 Finland; 4 Molecular and Integrative Biosciences Research Program, Faculty of Biological and Environmental Sciences, University of Helsinki, University of Helsinki, Viikinkaari 1, P.O. Box 65, Helsinki, 00014 Finland; 5 Department of Genomics, Laboratory Division, Turku University Hospital, Kiinamyllynkatu 10, Turku, 20520 Finland

**Keywords:** Prostate cancer, ANO7, Mitochondria, Lipid metabolism, MYC, OXPHOS, Glycolysis

## Abstract

**Background:**

Prostate cancer (PrCa) is a significant health concern, ranking as the second most common cancer in males globally. Genetic factors contribute substantially to PrCa risk, with up to 57% of the risk being attributed to genetic determinants. A major challenge in managing PrCa is the early identification of aggressive cases for targeted treatment, while avoiding unnecessary interventions in slow-progressing cases. Therefore, there is a critical need for genetic biomarkers that can distinguish between aggressive and non-aggressive PrCa cases. Previous research, including our own, has shown that germline variants in ANO7 are associated with aggressive PrCa. However, the function of ANO7 in the prostate remains unknown.

**Methods:**

We performed RNA-sequencing (RNA-seq) on RWPE1 cells engineered to express ANO7 protein, alongside the analysis of a single-cell RNA-sequencing (scRNA-seq) dataset and RNA-seq from prostate tissues. Differential gene expression analysis and gene set enrichment analysis (GSEA) were conducted to identify key pathways. Additionally, we assessed oxidative phosphorylation (OXPHOS), glycolysis, and targeted metabolomics. Image analysis of mitochondrial morphology and lipidomics were also performed to provide further insight into the functional role of ANO7 in prostate cells.

**Results:**

ANO7 expression resulted in the downregulation of metabolic pathways, particularly genes associated with the MYC pathway and oxidative phosphorylation (OXPHOS) in both prostate tissue and ANO7-expressing cells. Measurements of OXPHOS and glycolysis in the ANO7-expressing cells revealed a metabolic shift towards glycolysis. Targeted metabolomics showed reduced levels of the amino acid aspartate, indicating disrupted mitochondrial function in the ANO7-expressing cells. Image analysis demonstrated altered mitochondrial morphology in these cells. Additionally, ANO7 downregulated genes involved in fatty acid metabolism and induced changes in lipid composition of the cells, characterized by longer acyl chain lengths and increased unsaturation, suggesting a role for ANO7 in regulating lipid metabolism in the prostate.

**Conclusions:**

This study provides new insights into the function of ANO7 in prostate cells, highlighting its involvement in metabolic pathways, particularly OXPHOS and lipid metabolism. The findings suggest that ANO7 may act as a key regulator of cellular lipid metabolism and mitochondrial function in the prostate, shedding light on a previously unknown aspect of ANO7’s biology.

**Supplementary Information:**

The online version contains supplementary material available at 10.1186/s12964-025-02081-7.

## Background

Prostate cancer (PrCa) is the second most prevalent cancer in males worldwide [1]. Genetic determinants are estimated to contribute significantly to PrCa risk, accounting for up to 57% of the associated risk [2]. The primary clinical challenge in managing PrCa is the early identification and targeted treatment of aggressive cases while avoiding overtreatment in indolent cases. Therefore, there is a pressing need for genetic biomarkers that can effectively differentiate between aggressive and non-aggressive PrCa cases.

Genome-wide association studies (GWAS) and extensive meta-analyses have identified 451 distinct risk variants associated with prostate cancer, including four notable variants in the anoctamin 7 (*ANO7*) gene [3]. Recent studies in African population have further linked stop-gain, protein truncating, and structural variants in *ANO7* to an increased risk of PrCa [4, 5]. Our previous research has demonstrated a significant association between single-nucleotide polymorphisms (SNPs) in the *ANO7* gene and the risk of aggressive prostate cancer [6]. Furthermore, individuals carrying the rs77559646 variant show enhanced responsiveness to docetaxel treatment [7]. Notably, homozygous carriers of the rs77559646 experience a complete loss of ANO7 protein expression within the prostate tissue [8]. Our findings have shed light on an additional layer of intricacy in the regulation of ANO7 expression within the prostate, as evidenced by the confinement of ANO7 mRNA to the nucleus [9]. The transition of prostate cancer from localized to metastatic stages is further highlighted by the documented diminution and eventual loss of ANO7 expression [10]. ANO7 is prominently expressed within the luminal cells of the human prostate. As a member of the anoctamin family, ANO7 belongs to a family of calcium-activated chloride channels [11, 12], and notably, possesses phospholipid scramblase activity [13]. Despite extensive evidence linking ANO7 variants to PrCa, the precise functional role of ANO7 in the prostate is still unknown. Recently, however, utilizing spatial transcriptomics from benign glands within PrCa tissue, we uncovered an association between ANO7 expression and lipid metabolism (Metsälä et al. 2025, accepted).

In light of our research objectives, we provide new insights into the functional role of ANO7 within prostate cells. We employed RNA-sequencing (RNA-seq) techniques on RWPE1 cells engineered to express ANO7 protein. This was coupled with a comprehensive analysis of a publicly accessible single-cell RNA-sequencing (scRNA-seq) dataset derived from normal prostate tissues. Following this, we performed differential gene expression analysis, followed by gene set enrichment analysis (GSEA). Interestingly, we found a downregulation of several metabolic pathways, particularly those associated with the oxidative phosphorylation (OXPHOS) pathway in ANO7-expressing cells. This finding correlates with our comprehensive assessments of OXPHOS and glycolysis, which unveiled a shift towards glycolysis in the ANO7-overexpressing cell line. Targeted metabolomics analyses corroborated these findings, revealing a reduction in the amino acid aspartate within the ANO7-expressing cell line, indicative of perturbed mitochondrial function. Image analysis further showed that the mitochondrial networks are less branched and more fragmented in the ANO7 cell line, demonstrating that mitochondrial morphology and function are affected by ANO7 expression. Lastly, since we found ANO7 to downregulate fatty acid metabolism gene sets and previous studies have shown ANO7 to function as a phospholipid scramblase [13], we also performed lipidomics analysis in the cell line. Interestingly, we found that ANO7 induces a shift towards longer acyl chain length and greater degree of unsaturation in several lipid classes. This opens up a new line of research regarding the function of ANO7 in regulating lipid metabolism in the prostate. Our research, for the first time, posits ANO7 as a key regulator of cellular lipid metabolism and mitochondrial function within the prostate.

## Methods

### Cell culture and generation of plasmids and cell lines

RWPE1 cells were ordered from ATCC and grown according to the distributor’s instructions in K-SFM media (Thermo Fisher Scientific, Waltham, MA, USA), supplemented with 5 ng/ml EGF, 50 µg/ml bovine pituitary extract, 100 U/ml penicillin, and 100 μg/ml streptomycin (Thermo Fisher Scientific).

To create stable RWPE1 cell lines overexpressing ANO7, we subcloned ANO7 from a pcDNA3-ANO7-V5-His plasmid, which was kindly provided by Professor Dr med. Karl Kunzelmann (University of Regensburg, Germany) [14]. We used primers containing EcoRI and XbaI sites to insert ANO7 in pLVX-IRES-mCherry vector (Takara Bio, Shiga, Japan). The plasmid DNA sequence was verified by Sanger sequencing. Lentiviruses were produced by the Genome Editing Core facility at Turku Bioscience (Turku, Finland). Transduction was performed in the presence of 10 µg/ml polybrene. Positive control cells transduced with the pLVX-IRES-mCherry vector, and ANO7 cells, transduced with pLVX-IRES-mCherry-ANO7 were selected and sorted with the same mCherry fluorescence gating with a Sony SH800 cell sorter (Sony Biotechnology, Japan). The transduced cell lines are hereafter referred to as RWPE1 control and ANO7 cells.

To create a green fluorescent protein (GFP)-tagged ANO7 plasmid we used In-Fusion cloning (Takara Bio) to replace the V5-His tag with GFP in the pcDNA3-ANO7-V5-His plasmid. The resulting pcDNA-ANO7-GFP plasmid was verified by Sanger sequencing.

### RNA sequencing analysis and gene set enrichment analysis

RWPE1 native, RWPE1 control, and ANO7 cells were seeded in 6-well plates at a density of 3 × 10^5^ cells per well. After 48 h, total RNA was isolated with TRIsure reagent (Meridian Bioscience, Cincinnati, OH, USA) according to the manufacturer’s instructions, with minor modifications that included an additional chloroform phase separation step and two additional washes with 75% ethanol. RNA concentration was measured with the Qubit RNA assay on a Qubit 2.0 fluorometer (Thermo Fisher Scientific), and RNA integrity was assessed with the Agilent High Sensitivity RNA ScreenTape assay on an Agilent 2200 TapeStation (Agilent Technologies, Santa Clara, CA, USA). All samples used for RNA sequencing had an RNA integrity number of ≥ 9.7. RNA sequencing was performed by Novogene Co, Ltd. Sequencing libraries were generated using NEBNext® Ultra™ RNA library Prep Kit for Illumina (NEB, USA) and sequenced on an Illumina platform, producing paired-end (2*150bp) raw reads. The raw data in FASTQ format were pre-processed using Cutadapt [15] with default parameters to remove low quality reads (Q < 33), adapter sequences, and Poly-N sequences. The resultant paired-end high quality reads were aligned to the Ensembl Human reference genome hg38 using STAR [16] with default parameters. FeatureCounts [17] was employed to count the number of reads mapped to each gene, including both novel and known genes. Differential expression analysis was conducted using DESeq2 [18] R package with default parameters, adjusted using Benjamini -Hochberg to control for multiple comparisons. Genes were filtered with a TPM cutoff value of 1 or greater in every sample to be included in the analysis. Differentially expressed genes with a padj-value < 0.05 were visualized with a heatmap using the hierarchical clustering method in a Python script. The gene annotation and enrichment analysis were performed with GSEA software (Broad Institute, version 4.3.2) using the Molecular Signatures Database (MSigDB) Hallmark gene sets [19–21]. The scRNA-seq [22] annotated data, were downloaded from the interactive data portal (https://www.prostatecellatlas.org/) in the h5ad format. SCANPY [23], a Python tool kit, was employed for downstream scRNA-seq analysis using the Python packages such as SciPy [24], NumPy [25], Pandas, and Matplotlib [26]. Visualization of cell clusters was performed using Leiden clustering. Luminal epithelial (LE)-KLK3 cells were grouped into ANO7-positive and ANO7-negative groups and visualized with UMAP plot. These clusters were used for differential gene expression employing Wilcoxon rank-sum test with a padj-value cutoff 0.05. Differentially expressed genes between groups were used for GSEA, as described above. Dot plots were generated using ggplot2 [27] (https://ggplot2.tidyverse.org) package in R. The web version of Correlation AnalyzeR [28] was used to determine ANO7 gene expression correlation in normal prostate samples from the ARCHS4 [29] database and to perform correlation-based gene set enrichment analysis (corGSEA) using the hallmark gene set.

### Western blotting

The cells were washed three times with ice-cold PBS and scraped into a 1% CHAPS lysis buffer (30 mM Tris–Cl pH 8, 150 mM NaCl and 1% CHAPS) with cOmplete EDTA-free Protease Inhibitor Cocktail (Roche, Indianapolis, IN, USA). The lysates were incubated on ice for 30 min and then centrifugated at 13,000 × g for 20 min. Prostate tissue samples were snap-frozen in liquid nitrogen, then homogenized in ice-cold PBS using an Ultra-Turrax homogenizer (IKA, Staufen, Germany). The homogenate was mixed 1:1 with 2X 1% CHAPS lysis buffer and incubated on a rotating platform for 2 h at + 4°C. The prostate lysate was then centrifuged at 13,000 × g for 30 min. Protein concentrations were determined with the BCA Protein Assay Kit (Thermo Fisher Scientific). Proteins were separated with SDS-PAGE on a 4–15% Mini-PROTEAN TGX precast gel (Bio-Rad, Hercules, CA, USA) and transferred onto an Immobilon-P PVDF membrane (Millipore, Bedford, MA, USA). The membrane was incubated with rabbit anti-ANO7 primary antibody (HSPA078464, Atlas Antibodies) at 1:1000 dilution and goat anti-rabbit HRP-linked secondary antibody (Abcam, ab97080) at 1:10000 dilution. As a loading control, the membrane was reblotted with β-actin antibody (8H10D10, Cell Signaling Technology) diluted 1:1000, followed by horse anti-mouse HRP-linked secondary antibody (7076, Cell Signaling Technology) at 1:3000 dilution. Protein bands were detected using the WesternBright Quantum Western blotting detection kit (Advansta Inc., Menlo Park, CA, USA).

### Cellular bioenergetics measurements

Mitochondrial respiration and glycolysis were measured using Seahorse XF Analyzer (Agilent Technologies, Santa Clara, CA, USA) following the manufacturer’s protocol, briefly outlined below. For all assays, 4 × 10^4^ RWPE1 control and ANO7 cells per well were plated in a 96-well Seahorse assay plate, one day before the actual assay run, allowing them to adhere and stabilize in the culture plate.

The Agilent Seahorse XF Cell Mito Stress Test (Agilent Technologies) measures key parameters of mitochondrial function by directly assessing the oxygen consumption rate (OCR) of the cells. Complete media were removed from the cells one hour prior to the Mito Stress Test. The cells were then washed with the basal Seahorse Assay Medium supplemented with 10 mM glucose, 1 mM glutamine, and 1 mM pyruvate. After washing, cells were incubated for one hour in the basal Seahorse assay medium at 37 °C in an incubator without CO_2_, allowing them to adapt to the assay conditions. Following the measurement of baseline OCR, 1.5 µM oligomycin (complex V / ATP synthase inhibitor), 2 µM carbonyl cyanide p-[trifluoromethoxy]-phenylhydrazone (FCCP, a mitochondrial uncoupler), and 0.5 µM combination of rotenone (complex I / NADH dehydrogenase inhibitor) and antimycin A (complex III / cytochrome c reductase inhibitor) solutions were sequentially added to each well to determine the ATP coupled respiration, maximum respiration, and non-mitochondrial oxygen consumption rates, respectively. The maximal respiratory rate was calculated as the difference between the OCR after FCCP addition and the lowest OCR reached after oligomycin addition.

The Agilent Seahorse XF Mito Fuel Flex Test (Agilent Technologies) is a modified version of the mitochondrial stress test described above and was used to evaluate the effect of different substrates on maximal respiratory capacity by measuring the rate of oxidation of individual substrates (glucose, glutamine, or long-chain fatty acid) in cells. The pathway inhibitors used were 3 µM BPTES (an inhibitor of glutamine oxidation, which blocks glutaminase, converting glutamine to glutamate), 4 µM Etomoxir (an inhibitor of long-chain fatty acid oxidation, which blocks carnitine palmitoyl transferase 1 alpha), and 2 µM UK5099 (an inhibitor of glucose oxidation, which blocks the action of the mitochondrial pyruvate carrier (MPC) that transports pyruvate to mitochondria). The cells were treated with a single inhibitor followed by the mitochondrial stress test inhibitors to calculate the dependency of each pathway.

XF Glycolysis Stress Test (Agilent Technologies) was used to assess glycolytic functions. One hour prior to running the glycolysis stress test, complete media were removed, and cells were washed with the basal Seahorse assay medium supplemented with 1 mM glutamine. After that, the cells were incubated for one hour in a 37 °C incubator without CO_2_ before the initial measurement. After measuring the non-glycolytic acidification rate, 10 mM glucose (which is converted to pyruvate through glycolysis), 1.5 µM oligomycin, and 50 mM 2-deoxyglucose-glucose (a competitive inhibitor of hexokinase, the first enzyme in the glycolysis pathway) solutions were sequentially added to each well. These additions were used to determine the rate of glycolysis under basal conditions and maximum glycolytic capacity, respectively. Glycolysis was defined as the glucose-induced increase in the extracellular acidification rate (ECAR) and was calculated by subtracting non-glycolytic acidification from the highest ECAR measurement following the addition of glucose. Maximum glycolytic capacity was calculated as the difference between the highest ECAR measurement during non-glycolytic acidification and the highest ECAR measurement after the addition of oligomycin.

The data obtained from all Seahorse assays were normalized to cellular DNA content using the CyQUANT Cell Proliferation Assay Kit (Thermo Fisher Scientific) according to the manufacturer’s protocol. Fluorescence was measured at 480 nm using a VictorX4 multimode plate reader (PerkinElmer, Waltham, Massachusetts, USA). The Seahorse Wave Desktop Software (Agilent Technologies) was used to analyze the results.

### Determination of mitochondrial DNA copy number

Genomic DNA was isolated from cell pellets with the Cytiva Blood Genomic Prep Spin Kit (Thermo Fisher Scientific) according to the manufacturer’s instructions. DNA concentration was measured with a Nanodrop One spectrophotometer (Thermo Fisher Scientific). Mitochondrial DNA copy number was measured using the QX200 AutoDG Droplet Digital PCR System (ddPCR) (Bio-Rad) as described previously [30]. Briefly, the MT-ND1 (assay ID: dHsaCPE5029120 (FAM)) and EIF2C1 (assay ID: dHsaCP1000002 (HEX)) Bio-Rad assays were used to measure mtDNA and nDNA, respectively. The 20 µl ddPCR reactions included 1 ng DNA, 900 nM primers, 250 nM probes, ddPCR Supermix for probes (no UTP,) and 5U of HindIII (NEB). Droplets were generated using a Bio-Rad automated droplet generator. PCR cycling conditions were as follows: enzyme activation 10 min at 95°C, followed by 40 cycles of denaturation at 94°C for 30 s followed by annealing and extension at 60°C for 1 min. Lastly, an enzyme deactivation step at 98° C for 10 min was performed. After an overnight incubation at 4°C, the droplets were read with the droplet reader, and absolute copy number was determined with QuantaSoft software as copies/µl.

### Confocal microscopy

For live cell imaging, RWPE1 control and ANO7 cells were seeded in 35 mm glass bottom dishes (Ibidi, Germany) at a density of 2 × 10^5^ cells per well. After 48 h of plating, cells were stained with 500 nM MitoTracker Deep Red (Thermo Fisher Scientific) and 10 µg/ml Hoechst (Thermo Fisher Scientific) at 37 °C for 30 min following the manufacturer's instructions. The cells were then washed three times with PBS, and fresh growth media were added and imaged. For fixed cell imaging, native RWPE1 cells were seeded in 12-well plates with coverslips at a density of 2 × 10^5^ cells per well. After 24 h, the cells were transfected with ANO7-GFP and GFP plasmids at a concentration of 1 µg per well using Lipofectamine 3000 Transfection Reagent (Thermo Fisher Scientific), following the manufacturer’s instructions. After an additional 24 h, the media were replaced with fresh media, and the cells were stained with MitoTracker Deep Red and Hoechst, as described above. The stained cells were then washed three times with PBS, fixed with 4% paraformaldehyde for 10 min at room temperature, and washed three times with PBS. Finally, the coverslips were mounted onto slides with VECTASHIELD Antifade Mounting Medium (Vector Laboratories, Inc., CA, USA), following the manufacturer’s instructions, and stored at + 4 °C until analysis.

Live-cell and fixed-cell imaging was performed using a 3i Marianas system with a Yokogawa CSU-W1 scanning unit on an inverted Zeiss AxioObserver Z1 microscope, controlled by SlideBook 6 software (Intelligent Imaging Innovations, Göttingen, Germany). A 100X 1,46 NA oil immersion objective was used for imaging. Images were acquired with a Prime BSI sCMOS camera (Teledyne Photometrics, Tucson, USA). Hoechst was visualized using a 405 nm laser for excitation and an emission window at 425–470 nm; GFP was visualized using a 488 nm laser for excitation and an emission window of 510–540 nm; and MitoTracker was visualized using a 640 nm laser for excitation and an emission window at 670–710 nm. The microscope stage was maintained at 37 °C with humidified 5% CO_2_ air during live-cell imaging.

### Electron microscopy

Cells were fixed with 5% glutaraldehyde in s-collidine buffer for a minimum of 48 h and postfixed with reduced osmium (1:1 mixture of 1% osmium tetroxide and 1.5% potassium ferrocyanide). After osmication, cells were dehydrated with ethanol and flat-embedded using the Fluka Epoxy Embedding Medium kit (Sigma-Aldrich). Thin sections were cut to a thickness of 70 nm using an EM UC7 ultramicrotome (Leica Microsystems GmbH, Germany). The sections were stained with 1% uranyl acetate and 0.3% lead citrate. The stained sections were examined using a JEM-1400 Plus transmission electron microscope (JEOL Ltd, Japan) operated at 80 kV acceleration voltage, and images were recorded with a Quemesa camera (EMSIS GmbH, Germany).

### Image analysis

The mitochondrial networks, branching, and morphology were analyzed and quantified from confocal microscopy images using the Mitochondria-Analyzer plugin in the ImageJ interface (NIH, Bethesda, MD, USA), following the method previously described [31]. For live-cell analysis, three experiments per group was performed and 10 image fields per experiment were used to analyze the mitochondrial network. In total, 30 mitochondria from control and ANO7 cells were analyzed. For fixed cells, eight slides per group and 10 image fields per slide were used to count the mitochondrial network. In total, 60 mitochondria were analyzed from control and ANO7 cells.

Additionally, electron microscopy images were used to measure mitochondrial length and area using ImageJ software [32]. A total of 100 mitochondria from control and ANO7 cells were quantified. Colocalization analysis was performed using the ImageJ plugin Coloc 2.

### Proliferation assay

RWPE1 control and ANO7 cells were seeded in 96-well plates at density of 5000 cells per well. Twenty-four hours after plating, 20 µM TFB-TBOA (Tocris Bioscience, Bristol, UK) was added to the cells. The drug (TFB-TBOA) was renewed every other day until the measurement to ensure continuous exposure throughout the experiment. After growing the cells for 6 days, cell proliferation was measured using Cell Proliferation Kit II (XTT) (Roche) according to the manufacturer's protocol. Absorbance was measured at 450nm using a VictorX4 multimode plate reader (PerkinElmer, Waltham, Massachusetts, USA).

### Metabolomics

RWPE1 control and ANO7 cells were plated in 10 cm dishes at a density of 5X10^5^ cells per dish. After 24 h the media was replaced with fresh media. When the number cell counts reached approximately 2 million, cells were extracted in liquid chromatography-mass spectrometry (LC–MS) extraction buffer composed of methanol/acetonitrile/MQH_2_O (40:40:20 by volume). Samples were stored at −80 °C until analysis. Mass spectrometry analysis was conducted by the FIMM Metabolomics Unit (FIMM-Meta) at the University of Helsinki. The samples were run on a Waters Acquity UPLC system coupled to a Xevo-TQS triple quadrupole mass spectrometer equipped with an electrospray ionization probe (Waters Corporation, Milford, MA, USA) as described [33]. An Atlantis dC18 (2.0 × 100 mm, 3-µm particles) reversed-phase analytical column from Waters (M/s Milford, MA, USA) was used as a chromatographic separation column. Data analysis was performed with MetaboAnalyst 5.0 by the FIMM Metabolomics Unit (FIMM-Meta) at the University of Helsinki.

### Lipidomics

Cellular lipids were analyzed at HiLIPID, University of Helsinki. Lipids were extracted using the Folch method [34] and dissolved in chloroform/methanol (1:2 by volume). Prior to mass spectrometry, internal standards (phosphatidylcholines PC 14:1/14:1 and PC 20:1/20:1, phosphatidylethanolamines PE 14:1/14:1 and PE 20:1/20:1, phosphatidylserine PS 14:1/14:1, phosphatidylinositol PI 16:0/16:0, sphingomyelin SM 18:1;O2/17:0, and triacylglycerol TG 20:0/20:0/20:0, from Avanti Polar Lipids, Merck, or prepared in house) and 1% NH_4_OH were added. The samples were infused to a triple quadrupole mass spectrometer 3Q-MS (Agilent 6410 Triple Quad LC/MS; Agilent Technologies, Santa Clara, CA) with a syringe pump (flow rate 10 µl/min) and spectra were recorded using positive and negative ionization and lipid class specific detection modes. Most phospholipids were detected using head group specific parent (P) or neutral loss (NL) scans: PC, PC O (PC alkyl-acyl species) and SM by P184, PE by NL141, PS by NL87, and PI by P241. In addition, PE P (PE alkenyl-acyl species) were detected as MS- ions (structures confirmed by alkenyl chain specific scans), and TGs as (M + NH_4_) + ions from MS + scan.

The presence of polyunsaturated fatty acids (PUFAs), such as arachidonic acid (20:4n-6), in the phospholipid species that were elevated in the ANO7 samples was confirmed by gas chromatography–mass spectrometry GC–MS (Shimadzu GCMS-QP2010 Ultra, Shimadzu, Kyoto, Japan) and by 3Q-MS (Agilent 6490 Triple Quad LC/MS; Agilent Technologies, Santa Clara, CA) using negative-mode precursor ion scans for acyl chains released from PE, PI, PS, and PC formate adducts (Figure S1, Additional file 1).

Lipid class and species concentrations of each sample were normalized to protein concentration (pmol/mg) and to total lipid (mol%). Multivariate comparisons of lipid species mol% profiles were performed with principal component analysis (PCA) in GraphPad Prism (v.9.3.1) for each lipid class. Statistical analysis was performed in MetaboAnalyst on log transformed and auto scaled values. The heat maps were generated in GraphPad Prism.

### Statistics

Statistical analysis was conducted using the GraphPad Prism (v.9.3.1) software, unless otherwise noted. Statistical differences among multiple groups were determined using one-way ANOVA with Tukey’s post hoc test. Differences between two groups were assessed with a two-tailed Student's t-test. A *P* value of < 0.05 was considered statistically significant.

## Results

### Metabolic pathways are altered by ANO7

**Fig. 1:**
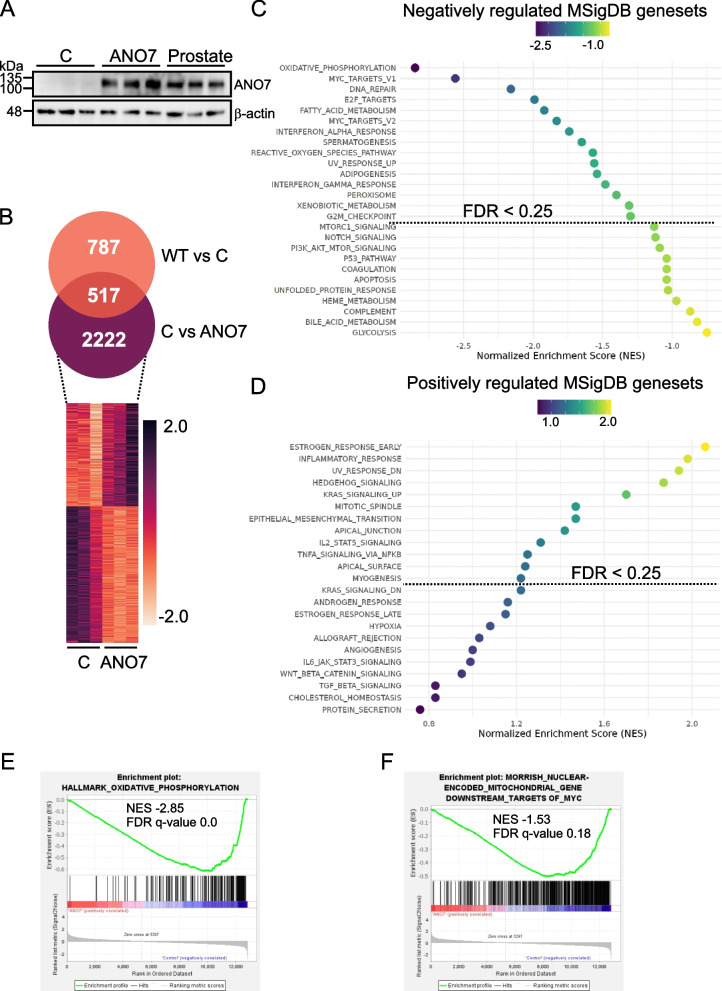
ANO7 alters gene expression in metabolic pathways. **A** ANO7 protein expression in the ANO7 overexpressing cell line was verified by western blotting and compared to ANO7 expression in three prostate samples obtained from patients undergoing cystectomy (*n* = 3). **B** RNA sequencing was performed in RWPE1 control cells and cells overexpressing ANO7 (*n* = 3). The differentially expressed genes with a padj-value < 0.05 are visualized by a heat map. **C** The dot plot shows what MSigDB Hallmark gene sets are negatively affected by ANO7 expression. **D** The dot plot shows MSigDB Hallmark gene sets affected positively by ANO7 expression. **E** An enrichment plot showing the OXPHOS genes being mainly downregulated within this dataset. **F** The enrichment plot shows that nuclear-encoded mitochondrial genes downstream of MYC are downregulated in ANO7 expressing cells. The dotted lines in (**C**,**D**) show the gene sets with an FDR q-value below 0.25

The cellular functions for ANO7 are currently unknown. Germline variants in ANO7 have been linked to aggressive PrCa and the rs77559646 variant leads to loss of ANO7 protein [6, 8]. Moreover, a reduced expression of ANO7 is a predictor of poor prognosis in PrCa [10]. Therefore, we hypothesized that ANO7’s role in PrCa is important in early-stage disease and benign tissue. The processes that ANO7 regulates in benign tissue could offer clues to why it is lost during PrCa progression. Based on our observations, ANO7 expression is low or absent in commercially available prostate epithelial cell lines. To investigate its functions, we utilized a lentiviral vector to transduce normal prostate epithelial cells (RWPE1) with cDNA encoding for ANO7. Protein lysates from the resulting control and ANO7 cell lines were analyzed and ANO7 protein expression in the ANO7 cell line was compared to three prostate samples obtained from patients undergoing cystectomy (Fig. 1A). The ANO7 protein level in the cell line was at a similar level to endogenous levels when normalized to β-actin. To explore the cellular functions for ANO7, we performed RNA-sequencing (RNA-seq) and gene set enrichment analysis (GSEA) using hallmark gene sets from The Molecular Signatures Database (MSigDB) to identify differentially expressed and enriched genes. In RWPE1 cells overexpressing ANO7, we identified 2222 genes that were differentially expressed, which were not among the genes differentially expressed between control cells and untransfected wt cells (Fig. 1B). Fifteen pathways were negatively regulated by ANO7 expression with a false discovery rate (FDR) q-value below 0.25: oxidative phosphorylation, MYC targets V1 and V2, DNA repair, E2F targets, fatty acid metabolism, interferon alpha response, spermatogenesis, reactive oxygen species pathway, UV response up, adipogenesis, interferon gamma pathway, peroxisome, xenobiotic metabolism, and G2M checkpoint (Fig. 1C). Twelve pathways were positively regulated by ANO7 expression with an FDR q-value below 0.25: including: estrogen response early, inflammatory response, UV-response dn, hedgehog signaling, KRAS signaling up, mitotic spindle, epithelial mesenchymal transition, apical junction, IL2 STAT signaling, TNFA signaling via NFKB, apical surface, and myogenesis (Fig. 1D). An interesting finding from the RNA-seq experiment was that mitochondrial genes involved in oxidative phosphorylation (OXPHOS) were enriched in RWPE1 cells overexpressing ANO7 (Fig. 1E). While the majority of OXPHOS genes were downregulated in ANO7 cells, a small proportion were among the highest upregulated genes (Figure S2, Additional file 1). These upregulated genes are encoded by the mitochondrial genome. The increase in mitochondrial transcripts could indicate an increased copy number of mitochondria. To investigate this, we measured mtDNA using ddPCR, but no significant changes were observed (Figure S3A, Additional file 1). This suggests that the increase in mitochondrial OXPHOS transcripts and the decrease in nuclear-encoded OXPHOS transcripts are due to transcriptional events. Intriguingly, both the OXPHOS and MYC pathways were among the most significantly negatively regulated pathways, and MYC is a known regulator of nuclear-encoded OXPHOS genes [35, 36]. Therefore, we used GSEA to investigate whether nuclear-encoded mitochondrial genes downstream of MYC are also negatively regulated in ANO7 cells. Indeed, using a gene set for nuclear-encoded mitochondrial genes downstream of MYC [36], we found that MYC signaling could explain the downregulation of mitochondrial genes observed in the ANO7 cells (Fig. 1F).

**Fig. 2:**
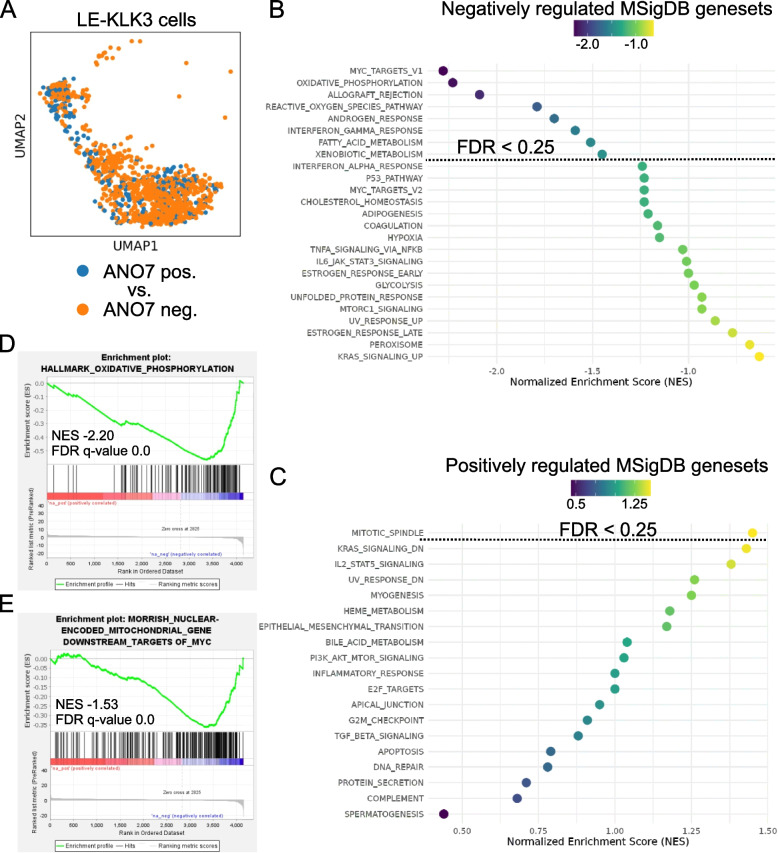
Differential expression and GSEA analysis on single cell RNA-seq from benign prostate tissue reveal that MYC signaling and oxidative phosphorylation is negatively affected by ANO7. **A** The LE-KLK3 cells of benign prostate tissue were divided into ANO7 positive and negative groups. Thereafter differential gene expression and gene set enrichment analysis was performed. **B** The dot plot shows MSigDB hallmark gene sets that were negatively correlating with ANO7 expression. **C** Gene set enrichment analysis of MSigDB Hallmark gene sets showed that only the mitotic spindle gene set is positively correlating with ANO7 expression. **D** The OXPHOS gene set enrichment plot shows that genes in this pathway are mostly downregulated in ANO7 expressing cells. **E** An enrichment plot showing that nuclear-encoded mitochondrial genes downstream of MYC are downregulated in prostate luminal epithelial KLK3 cells expressing ANO7. The dotted lines in (**B**,**C**) show the gene sets with an FDR q-value below 0.25

To validate the in vitro findings, we utilized a publicly available single cell RNA-seq data set performed on normal human prostate cells [22]. Since ANO7 is exclusively expressed in luminal epithelial cells of the prostate, we focused on LE-KLK3-positive cells from this dataset and divided them into ANO7 positive and negative groups (Fig. 2A). We then performed differential expression analysis between LE-KLK3-positive luminal cells expressing ANO7 and those not expressing ANO7. Intriguingly, gene set enrichment analysis (GSEA) revealed a significant overlap between the pathways enriched in our in vitro experiment and those identified in the in vivo analysis. Specifically, seven out of nine pathways enriched in the in vitro experiment also showed enrichment in the in vivo data. Pathways negatively regulated by ANO7 expression with an FDR q-value below 0.25 included MYC targets V1, oxidative phosphorylation, allograft rejection, reactive oxygen species pathway, androgen response, interferon gamma response, fatty acid metabolism, and xenobiotic metabolism (Fig. 2B). Only the mitotic spindle pathway was positively regulated by ANO7 expression with an FDR q-value below 0.25 (Fig. 2C). Importantly, we corroborated our in vitro findings that ANO7 negatively affects the oxidative phosphorylation pathway using this dataset (Fig. 2D), and showed that nuclear-encoded mitochondrial genes downstream of MYC are negatively regulated by ANO7 (Fig. 2E). Additionally, we employed the Correlation AnalyzeR tool to evaluate how ANO7 expression affects signaling pathways by performing correlation-based gene set enrichment analysis (corGSEA) with prostate samples from the ARCHS4 database. Consistent with our findings, ANO7 expression negatively correlated with oxidative phosphorylation, which was among the most significantly negatively regulated pathways (NES −4.4, padj-value 0.003) (Table S1, Additional file 1).

### ANO7 regulates mitochondrial function

**Fig. 3:**
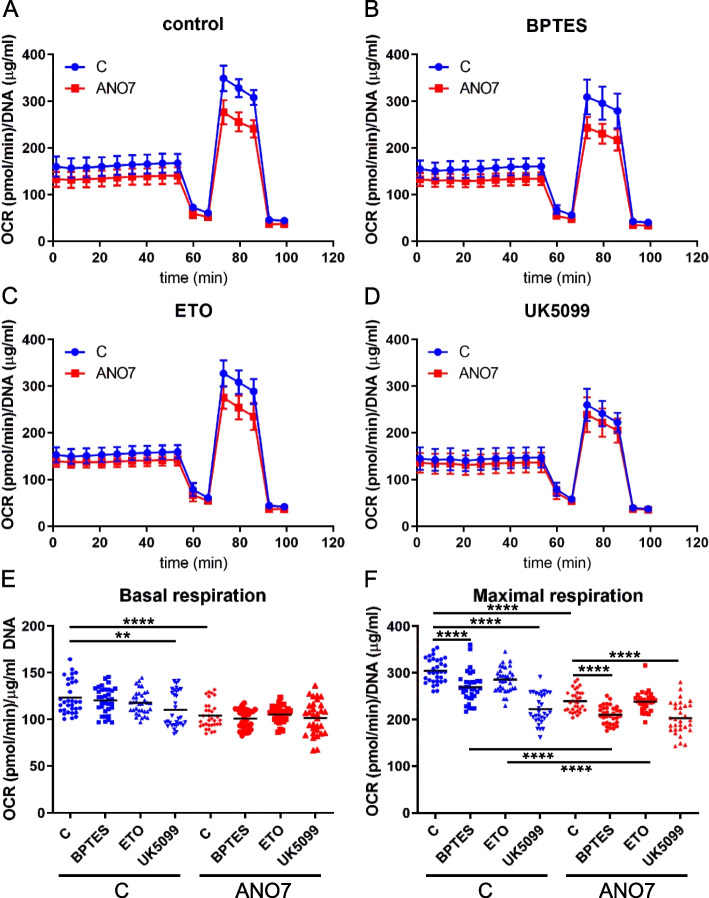
Oxidative phosphorylation is reduced in cells expressing ANO7. **A** Agilent Seahorse Mito Stress Test performed in RWPE1 control and ANO7 cells. **B** The Mito Stress Test was performed in the presence of the glutaminolysis inhibitor BPTES. **C** Etomoxir was used to inhibit usage of long chain fatty acids for OXPHOS in combination with the Mito Stress Test. **D** Inhibition of pyruvate import to mitochondria by UK5099 was used to study the effect of the glucose pathway for OXPHOS. **E** and **F** Quantification of basal and maximal respiration as obtained in (**A**-**D**). **, *p* < 0.01, ****, *p* < 0,0001

**Fig. 4:**
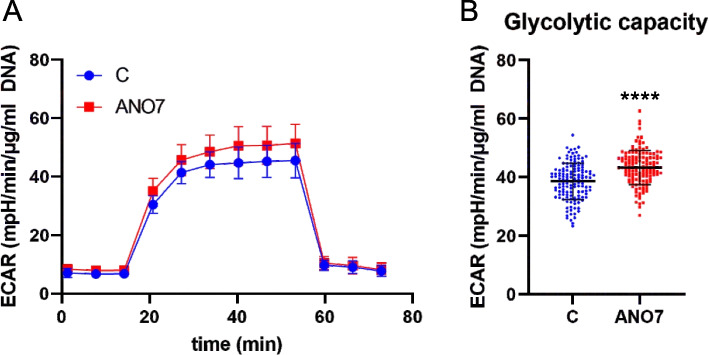
Glycolytic capacity is increased in ANO7 cells. **A** The glycolysis stress test was performed with RWPE1 control and ANO7 cells. **B** Glycolytic capacity quantified from (**A**). ****, *p* < 0,0001

Given that gene set enrichment analyses from both our in vitro data and publicly available patient data indicated that ANO7 negatively regulates OXPHOS, we utilized the Agilent Seahorse XF 96e Analyzer along with the Mito Stress Test and Mito Fuel Flex Test to measure OXPHOS and the utilization of different fuel sources (glucose, glutamine, and long-chain fatty acids) in the cells. We observed a reduction in both basal and maximal respiratory capacity in ANO7-overexpressing cells compared to control cells (Fig. 3A, E, F). Inhibition of glutamine usage for OXPHOS reduced maximal respiration in both the control and ANO7 cell lines compared to the no-drug condition (Fig. 3B, F). However, a significant difference between the cell lines persisted, suggesting that reduced glutamine usage does not fully explain the decreased OXPHOS observed in the ANO7 cell line. Inhibition of long-chain fatty acid utilization had no significant effect on OXPHOS in these cells (Fig. 3C, F). Treatment with UK5099, an inhibitor of glucose metabolism that blocks pyruvate import into mitochondria, led to reduced basal and maximal respiration in both control and ANO7 cell lines (Fig. 3D, E, F). Notably, there were no significant differences in basal or maximal respiration between control and ANO7 cells under UK5099 treatment. This finding indicates that the reduced basal and maximal respiration observed in ANO7 cells is likely due to decreased usage of glucose-derived pyruvate for OXPHOS. Given the observed reduction in energy production from OXPHOS, we hypothesized a possible metabolic shift towards increased glycolysis. Indeed, the Glycolysis Stress Test revealed increased glycolytic capacity in ANO7-overexpressing cells (Fig. 4A, B). The changes observed in cellular bioenergetics could be a result of differences in the mitochondrial respiratory complexes. We utilized an antibody cocktail containing antibody markers for each of the five complexes and measured their abundance by western blotting. However, we could not observe any significant changes control and ANO7 cells (Figure S3B, Additional file 1).

### ANO7 regulates mitochondrial morphology

**Fig. 5:**
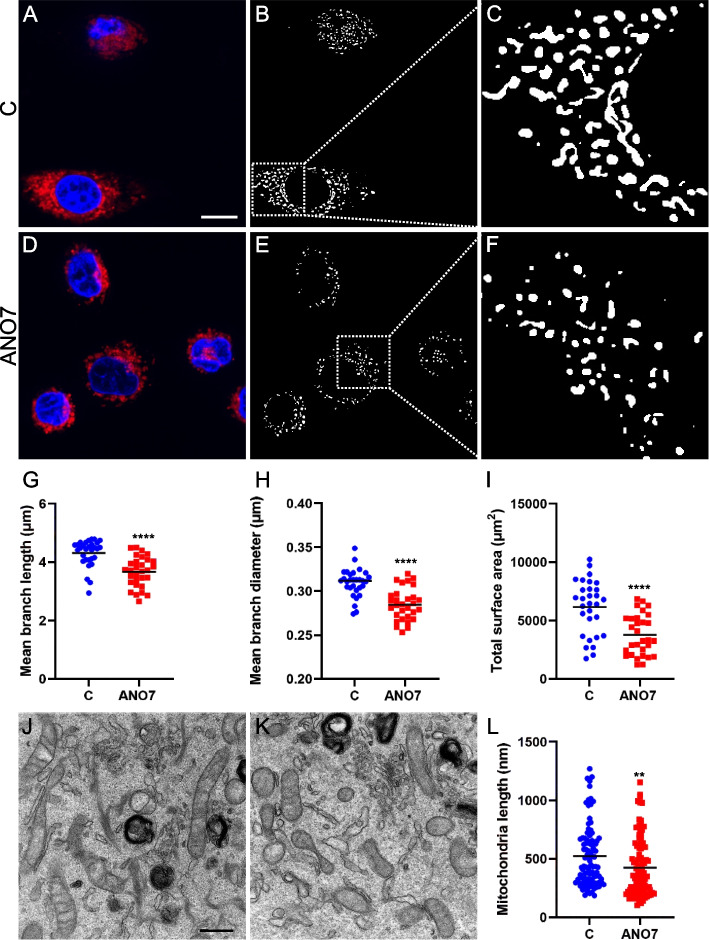
ANO7 expression induces mitochondrial fragmentation. **A** Representative confocal microcopy image of control cells stained with MitoTracker Deep Red and Hoechst nuclear dye. The scale bar indicates 10 µm. **B** The (**A**) image that has been processed for quantification according to methods described in the materials and methods section. **C** Magnification of a part of image (**B**). **D** A representative example of ANO7 cells stained with MitoTracker Deep Red and Hoechst. **E** The (**D**) image that has been processed and thresholded for quantification of mitochondrial networks. **F** A magnification of the mitochondrial networks in image (**E**). **G**-**I** Quantification of MitoTracker Deep Red staining in control and ANO7 cells revealed reduced mitochondrial mean branch length (**G**), mean branch diameter (**H**), and total surface area (**I**). **J**, **K** Electron microscopy image of mitochondria in control (**J**) and ANO7 (**K**) cells. The scale bar indicates 500 nm. **L** Mitochondria length was quantified from the electron microscopy images. **, *p* < 0,01, ****, *p* < 0,0001

**Fig. 6:**
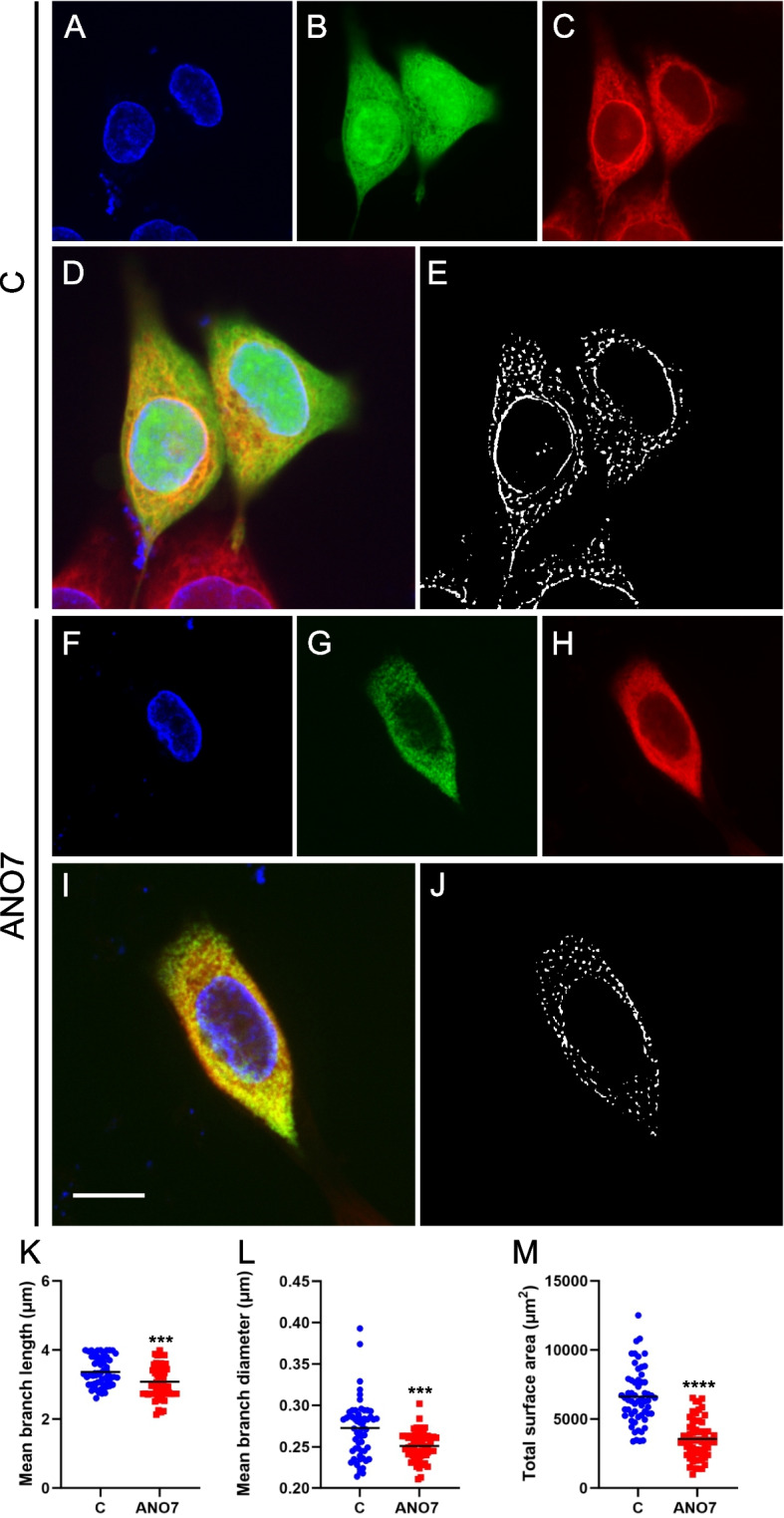
Transient ANO7 expression in RWPE1 cells leads to mitochondrial fragmentation. **A**-**C** A representative confocal image of native RWPE1 cells stained with Hoechst (**A**), transfected with GFP (**B**), and stained with MitoTracker Deep Red (**C**). **D** A merged image showing all treatments (**A**-**C**) together. **E** A processed image of (**C**) that was used to quantify the mitochondrial networks. **F**–**H** A representative confocal image of a native RWPE1 cell stained with Hoechst (**F**), transfected with ANO7-GFP (**G**), and stained with MitoTracker Deep Red (**H**). **I** A merged image visualizing all treatments (**F**–**H**) together. The scale bar indicates 10 µm. **J** MitoTracker Deep Red signal (**H**) that has been processed for the quantification of mitochondrial networks. The quantification of the mitochondrial networks revealed a decrease in mitochondrial mean branch length (**K**), mean branch diameter (**L**), and total surface area (**M**) in the ANO7-GFP transfected cells compared to GFP transfected control cells. ***, *p* < 0,001, ****, *p* < 0,0001

Mitochondrial dynamics and network morphology are crucial for mitochondrial function, quality control, and overall cell health, as well as for adaptation to stress [37]. Typically, healthy mitochondria are mobile and tubular, forming complex networks. In contrast, cells under significant stress or undergoing apoptosis often exhibit swollen and fragmented mitochondria, which is associated with disruptions in metabolism, membrane potential, ROS levels, and Ca^2^⁺ signaling [38]. Therefore, quantitative imaging-based assessment of mitochondrial morphology and dynamics is essential for understanding cellular physiology and pathophysiology. Using MitoTracker Deep Red staining followed by confocal microscopy, we observed that mitochondria in ANO7 cells were more fragmented compared to control cells. This fragmentation was characterized by significant changes in mean branch length, mean branch diameter, and total surface area (Fig. 5A-I). To validate these observations with higher resolution, we performed electron microscopy. The electron microscopy images confirmed that mitochondria in ANO7 cells were indeed more fragmented, with notable alterations in mitochondrial length (Fig. 5J-L). Since this result was obtained from a stable cell line overexpressing ANO7, we sought to investigate whether a similar phenotype could be observed after transient 24-h transfection. We compared ANO7-GFP transfected cells stained with Hoechst and MitoTracker Deep Red and compared them to GFP-transfected control cells (Fig. 6A-J). Interestingly, when quantifying the mitochondrial networks from these images we could see a significant decrease in the mean branch length, mean branch diameter, and total surface area of the ANO7-GFP transfected cells compared to GFP-transfected control cells (Fig. 6K-M). Interestingly, there seemed to be a significant overlap between the ANO7-GFP signal and the MitoTracker Deep Red signal (Fig. 6G-I). Colocalization analysis revealed a significant linear relationship, as shown by Pearson's and Spearman's correlation coefficients. This analysis, which included 23 ANO7-GFP positive cells, yielded an average Pearson's correlation coefficient of 0.74, reflecting a substantial linear overlap in pixel intensities between ANO7 and the mitochondrial marker. Similarly, the average Spearman's rank correlation coefficient was 0.76, indicating a high level of agreement in the relative rankings of these intensities. These results suggest that ANO7 indeed localizes close to mitochondria.

### Aspartate is a limiting factor for ANO7 cell proliferation

**Fig. 7:**
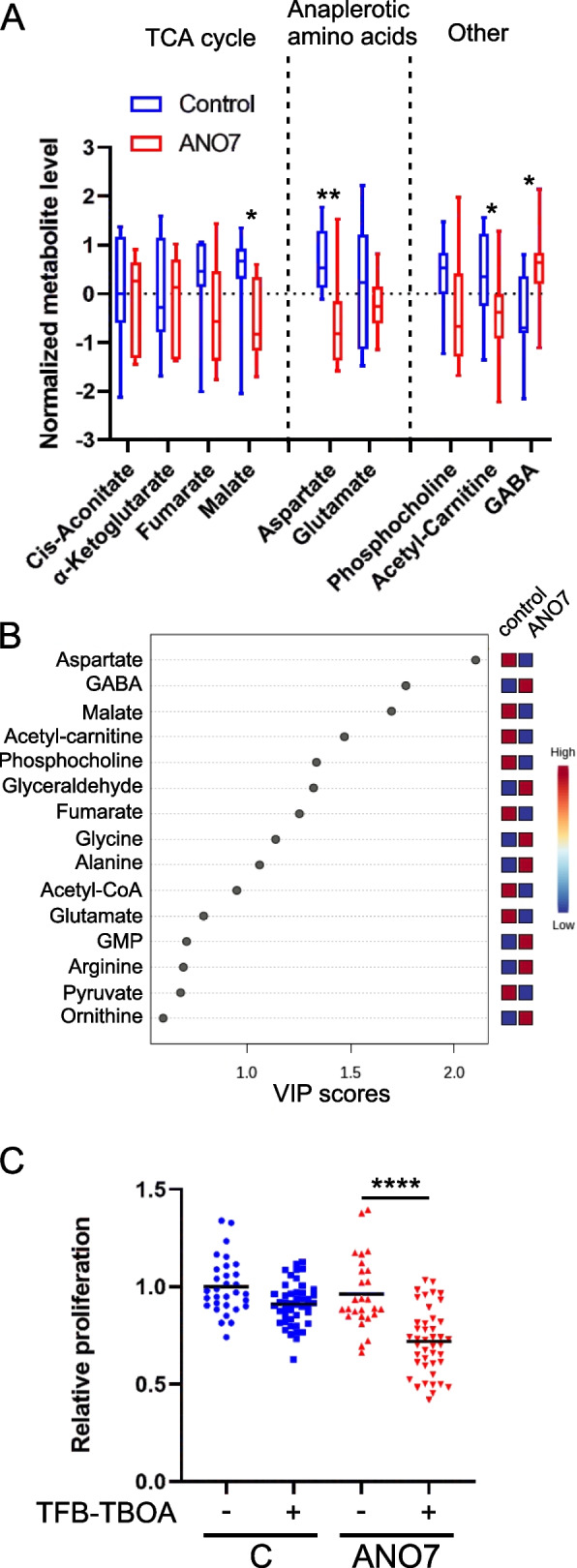
Metabolomics analysis revealed aspartate as a limiting factor for ANO7 cell growth. **A** Targeted metabolomics analysis was performed in RWPE1 control and ANO7 cells. The results are visualized with a boxplot (**B**) Partial Least Squares Discriminant Analysis was performed and the variable importance in projection plot shows the important features identified in descending order of importance. **C** RWPE1 control and ANO7 cells was allowed to grow in the absence or presence of 20 µM TFB-TBOA for six days to measure the impact of aspartate uptake inhibition on proliferation. *, *p* < 0,05 **, *p* < 0,01, ****, *p* < 0,0001

To unveil the specific metabolic effects of ANO7 overexpression, we conducted a targeted metabolite screen of control and ANO7 expressing cells. While no clear pattern of significantly altered levels of tricarboxylic acid (TCA) cycle intermediates was observed, several metabolites showed significant changes, indicating altered mitochondrial function in ANO7 cells. Notably, we observed significant reductions in malate, acetyl-L-carnitine, and aspartate levels, while γ-aminobutyric acid (GABA) levels were increased (Fig. 7A, B). The most pronounced change was a reduction in aspartate levels in ANO7 cells. Previous studies suggest that aspartate becomes a limiting factor when cells are exposed to mitochondrial electron transport chain inhibitors or hypoxia, as it is utilized for nucleotide synthesis under these conditions [39, 40]. To determine if aspartate limitation affects the growth of ANO7 cells, we assessed cell proliferation (viability) over six days with or without TFB-TBOA, an inhibitor of aspartate uptake (Fig. 7C). Under control conditions, there were no differences in proliferation between cell lines. However, in the presence of TFB-TBOA, ANO7 cells exhibited significantly slower growth compared to control cells, whereas the inhibition of aspartate uptake had no significant impact on the growth of control cells.

### ANO7 regulates phospholipid acyl chain length and degree of unsaturation

**Fig. 8:**
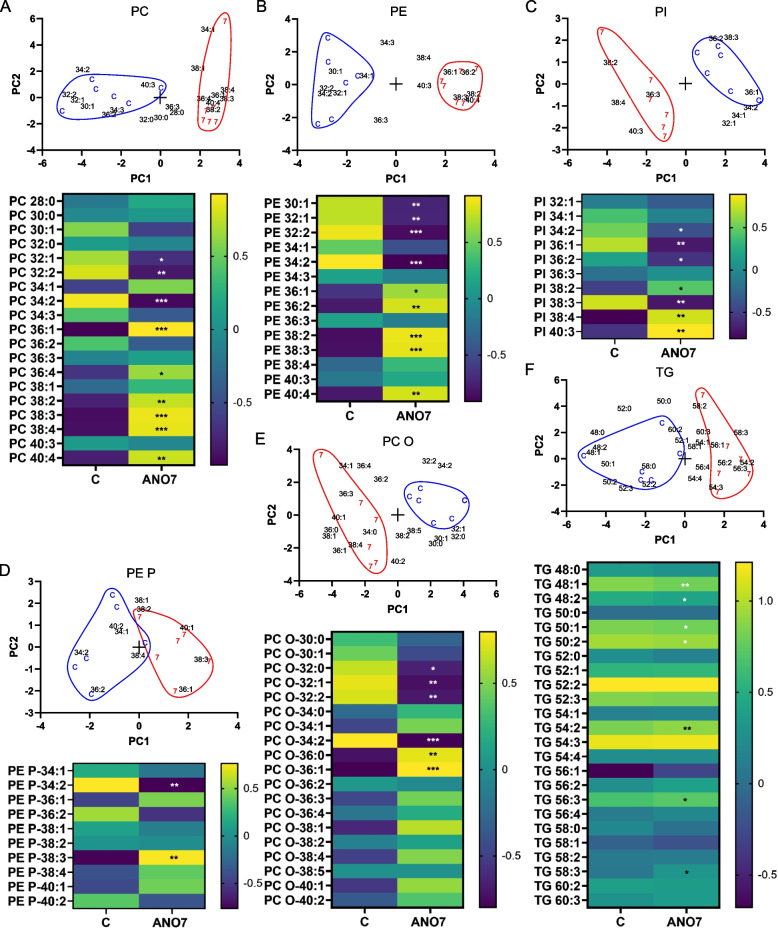
ANO7 expression modulates lipid species in prostate cells. PCA biplots of mass spectrometric lipidomes of control (**C**) and ANO7 overexpressing cells (7) using as loadings mol% data of (**A**) phosphatidylcholine diacyl species (PC), (**B**) phosphatidylethanolamine diacyl species (PE), (**C**) phosphatidylinositol species (PI), (**D**) phosphatidylethanolamine plasmalogen i.e. alkenyl-acyl species (PE P), (**E**) phosphatidylcholine alkyl-acyl species (PC O) and (**F**) triacylglycerol species (TG), all followed by a corresponding heatmap below (the color coded values in each cell of the heatmap are z scores, calculated as sample value – average value/standard deviation). *, *p* < 0,05 **, *p* < 0,01, ***, *p* < 0,001

As ANO7 has been shown to function as a phospholipid scramblase and our RNAseq data indicate downregulation of fatty acid metabolism genes, we utilized mass spectrometry to investigate whether ANO7 expression has any impact on the lipid class or species compositions. There were no significant differences in the total proportions of any of the studied phospholipid classes (PC, PC O, PE, PE P, PS, PI, SM) or TGs between the cells differently expressing ANO7 (Additional file 2). Next, PCA and heatmaps were used to reveal changes in the lipid species profiles for each analyzed lipid class (Fig. 8). Interestingly, the ANO7 samples contained significantly higher proportions of PC, PE, and PI species with longer acyl chains and a higher degree of unsaturation compared to the control cells. In addition, the PC O, PE P and TG species of ANO7 samples had longer acyl chains than the controls, whereas no significant species differences were found in SM and PS. Since there was a clear trend in the ANO7 samples towards an increase in polyunsaturated species with 3 or 4 double bonds, we studied their acyl chain assemblies further by using acyl chain specific MS/MS scans with 3Q-MS and determined the acyl chain structures by GC-MSD (Figure S1, Additional file 1). These analyses confirmed that the polyunsaturated species getting higher relative amounts in the ANO7 samples contained arachidonic acid 20:4n-6 (in species PC 36:4, PE 38:4 partly, PE P-38:4 and PI 38:4), but also mead acid 20:3n-9 (in species PE 38:3, PE 38:4 partly, PE P-38:3, PI 36:3, PI 38:3). The 20:4n-6 is a precursor of inflammatory lipid mediators, and high content of 20:3n-9 indicates that the cells had limited supply of essential PUFAs (e.g., linoleic acid 18:2n-6 or 20:4n-6), which is expected as the cells were grown in serum-free K-SFM media.

## Discussion

This study sheds light on the previously unexplored functional role of ANO7 in prostate cells with a particular focus on its impact on mitochondrial function. The research highlights the significant downregulation of genes associated with OXPHOS in ANO7-overexpressing prostate cells. The observed downregulation of OXPHOS-related genes suggests that ANO7 may play a role in modulating energy production within prostate cells. In addition to our data from the cell model, we were able to show that ANO7 expression correlates negatively with the OXPHOS pathway in both a scRNA data set and in prostate samples in the ARCHS4 database.

To gain a more comprehensive understanding of ANO7's impact on mitochondrial function, we assessed cellular respiration and glycolysis. We observed that ANO7-overexpressing cells exhibited a reduction in both basal and maximal respiratory capacity, indicating impaired OXPHOS. Additionally, we found that a metabolic shift towards glycolysis had occurred as glycolytic capacity was higher in ANO7-expressing cells, further emphasizing the change in mitochondrial function. The luminal cells of the prostate have a distinctive mitochondrial metabolism, which enables them to contribute to semen composition in a unique way. Luminal cells of the prostate exhibit an intriguing feature where they accumulate high levels of zinc ions. This accumulation is thought to have an inhibitory effect on mitochondrial aconitase, an enzyme responsible for the conversion of citrate to isocitrate in the TCA cycle. The net result of this inhibition is the retention of citrate within the luminal cells, which subsequently is secreted with the prostatic fluid. However, this metabolic adaptation comes at a cost. Due to the truncated TCA cycle, the energy production of prostate luminal cells is less efficient, making these cells more reliant on aerobic glycolysis [41]. Thus, ANO7 expression could modulate mitochondrial function in the RWPE1 cells to more reflect the situation observed in the luminal cells of the prostate.

In both our GSEA analysis on cells and on patient material, we encountered negative enrichment of the MYC signaling pathway and the OXPHOS pathway. Especially the OXPHOS transcripts encoded by nuclear genes were downregulated. MYC has been shown to stimulate nuclear encoded mitochondrial genes [35]. This is indicative of that MYC signaling could be perturbed in the ANO7 cells and could be the reason for lower OXPHOS in the cells. Interestingly, we found that the inhibition of the MPC with UK5099 reduced respiration in control cells to levels comparable to those seen in ANO7 cells. A recent study showed that MPC regulates cell fate in the prostate [42]. MPC inhibition affects basal to luminal cell differentiation negatively and positions MPC as a key regulator of prostate epithelial cell differentiation. Interestingly, UK5099 treatment reduced prostate luminal markers, while it increased basal cell markers, inflammatory and glycolytic genes. In our analysis, GSEA revealed a positive enrichment of Hallmark gene sets the inflammatory response, IL2 STAT5 signaling, and TNFA signaling via NFKB. Furthermore, we saw a positive enrichment of epithelial mesenchymal transition in the ANO7 cells. Taken together, our data indicates that although ANO7 is expressed in mature prostate epithelial cells, it may have an inhibitory role for prostate epithelial cell maturation by inhibiting glucose usage for OXPHOS. Moreover, androgen receptor regulated PrCa has been shown to transcriptionally upregulate MPC [43]. Inhibitory action of the MPC should therefore have a disadvantageous effect on PrCa and could potentially be a reason why ANO7 mutations and downregulation are observed in PrCa.

Furthermore, our study explored the impact of ANO7 on mitochondrial morphology. It was observed that the mitochondria in ANO7-overexpressing cells exhibited more fragmented mitochondrial networks, which were measured using image analysis of MitoTracker-stained cells and electron microscopy images. More fragmented mitochondrial networks are frequently observed in cells where a metabolic shift from OXPHOS to glycolysis has occurred [44]. Therefore, this further supports the notion that ANO7 affects mitochondrial function in the cells, which compensates by having a higher glycolytic capacity. Analysis of ANO7-GFP transfected cells revealed a significant overlap with the mitochondrial marker. This suggests that ANO7 resides close to mitochondria and thus possibly affects mitochondrial function directly.

The targeted metabolomics analysis uncovered specific metabolic changes associated with ANO7 expression, particularly a significant reduction in aspartate levels. Aspartate is an essential metabolite involved in various cellular processes, including nucleotide synthesis. The decrease in aspartate in ANO7-expressing cells suggests a potential limitation in nucleotide synthesis and further highlights the impact of ANO7 on cellular metabolism. As ANO7-expressing cells had a lower level of aspartate, we found that blocking the uptake of aspartate significantly hampered the growth of ANO7-expressing cells, while having no effect on control cells. The only TCA intermediate that showed a statistically significant change was malate. However, ANO7 cells were more reliant on an extracellular source of aspartate for their growth, which is indicative of a TCA cycle defect. In addition, we saw a reduction in acetyl-carnitine, which is indicative of reduced fatty acid import to mitochondria for β-oxidation [45]. The observed increase in GABA could reflect the possibility that ANO7 cells are utilizing the GABA shunt to bypass two steps in the TCA cycle to produce succinate from glutamate [46]. The GABA shunt has been shown to be important for castration-resistant prostate cancer cells as GABA promotes the nuclear localization of the androgen receptor [47].

The lipidome of the cells expressing ANO7 contained higher proportions of lipid species with long and polyunsaturated acyl chains, while at the same time having fewer species with short and saturated or monounsaturated acyl chains compared to the control cells. Although lipid scramblases dissipate membrane lipid asymmetry, participate in the synthesis of glycosylated lipids, and promote vesiculation [48–50], they have not been reported to alter the overall acyl chain assemblies of cellular phospholipids, as we found in this study. Therefore, the lipidome changes induced by ANO7 may be related to the marked convergent downregulation of MYC target and fatty acid metabolism genes that we detected in the RWPE1 cells and prostate tissue with high ANO7. When ANO7 overexpression downregulates MYC genes, as part of wide metabolic reprogramming, it also downregulates fatty acid synthesis and lipogenesis [51, 52]. The primary product of de novo fatty acid synthesis is palmitate 16:0, which is partly Δ9-desaturated to 16:1n-7 or elongated and desaturated to 18:1n-9. These acyl chains are used to synthesize 32 and 34 carbon phospholipid species with 1 or 2 double bonds. Thus, detecting lowered proportions of these phospholipid species in ANO7-expressing cells suggests that the supply of de novo synthesized fatty acids of the cells was decreased, and more exogenous long-chain PUFAs were incorporated into their phospholipids [53]. Since cancer cells have been found to synthesize de novo fatty acids in excess [54], and prostate cancer becomes aggressive with overexpression of fatty acid synthase [55], suppressing the levels of de novo fatty acids by elevated ANO7 may offer a mechanism to limit cancerous lipogenesis.

Since lipid mediators controlling inflammation are produced from PUFA precursors that phospholipases have cleaved from membrane phospholipids, the membrane PUFA contents modulate inflammation [56]. The PUFAs of the RWPE1 cells were identified to contain 20:4n-6, which is a precursor of pro-inflammatory lipid mediators [57], and 20:3n-9, which has also been proposed to be a lipid mediator precursor but with poorly known effects [58]. Thus, the increased supply of the PUFA precursors for the production of pro-inflammatory lipid mediators may have contributed to the upregulation of inflammatory response genes in the ANO7-expressing RWPE1 cells in this study. Based on animal experiments, increasing the supply of n-3 PUFA supplements with anti-inflammatory effects could alleviate this condition [59]. Furthermore, the higher ANO7 and increased polyunsaturated phospholipid levels may together promote the secretion of vesicles from the prostate epithelial cells, due to temporarily losing local membrane asymmetry by ANO7 and enhancing vesicle fission by increased membrane unsaturation [48, 60].

The insights into the functional role of ANO7 in the prostate, as elucidated in this study, offers promising opportunities for further research in the field of prostate function and cancer. Understanding the mechanisms by which ANO7 affects mitochondrial function and limits the de novo fatty acid levels may lead to the development of targeted therapies and the identification of potential biomarkers for aggressive prostate cancer cases. In conclusion, this study provides a compelling foundation for future investigations into the precise mechanisms through which ANO7 influences prostate cancer progression and the potential clinical applications of these findings in the diagnosis and treatment of prostate cancer.

## Conclusions

Previous research by our group and others has shown that *ANO7* variants are linked to aggressive PrCa. In the present study, we have provided new insights into ANO7 function in prostate cells and observed for the first time that MYC target genes and OXPHOS genes are downregulated in ANO7-expressing cells. Furthermore, we observed a metabolic shift from OXPHOS to glycolysis and reduced de novo lipogenesis.

## Supplementary Information

Supplementary Material 1.

Supplementary Material 2.

## Data Availability

No datasets were generated or analysed during the current study.

## Abbreviations

ANO7: Anoctamin 7
PrCa: Prostate cancer
GWAS: Genome-wide association studies
SNPs: Single-nucleotide polymorphisms
RNA-seq: RNA-sequencing
scRNA-seq: Single-cell RNA-sequencing
GFP: Green fluorescent protein
GSEA: Gene set enrichment analysis
OXPHOS: Oxidative phosphorylation
MSigDB: Molecular Signatures Database
LE: Luminal epithelial
OCR: Oxygen consumption rate
FCCP: Carbonyl cyanide p-[trifluoromethoxy]-phenylhydrazone
MPC: Mitochondrial pyruvate carrier
ECAR: Extracellular acidification rate
ddPCR: Droplet digital PCR
LC-MS: Liquid chromatography-mass spectrometry
PC: Phosphatidylcholines
PE: Phosphatidylethanolamines
PS: Phosphatidylserine
PI: Phosphatidylinositol
SM: Sphingomyelin
TG: Triacylglycerol
P: Parent
NL: Neutral loss
PUFAs: Polyunsaturated fatty acids
TCA: Tricarboxylic acid
GABA: γ-Aminobutyric acid
