# Supplementary material for: ANO7 expression in the prostate modulates mitochondrial function and lipid metabolism
Source: Cell Commun Signal. 2025 Feb 8;23:71. doi: 10.1186/s12964-025-02081-7 (PMC11807338; doi:10.1186/s12964-025-02081-7)
Supplement: Supplementary file 1 — Supplementary Material 1. [file 12964_2025_2081_MOESM1_ESM.docx]

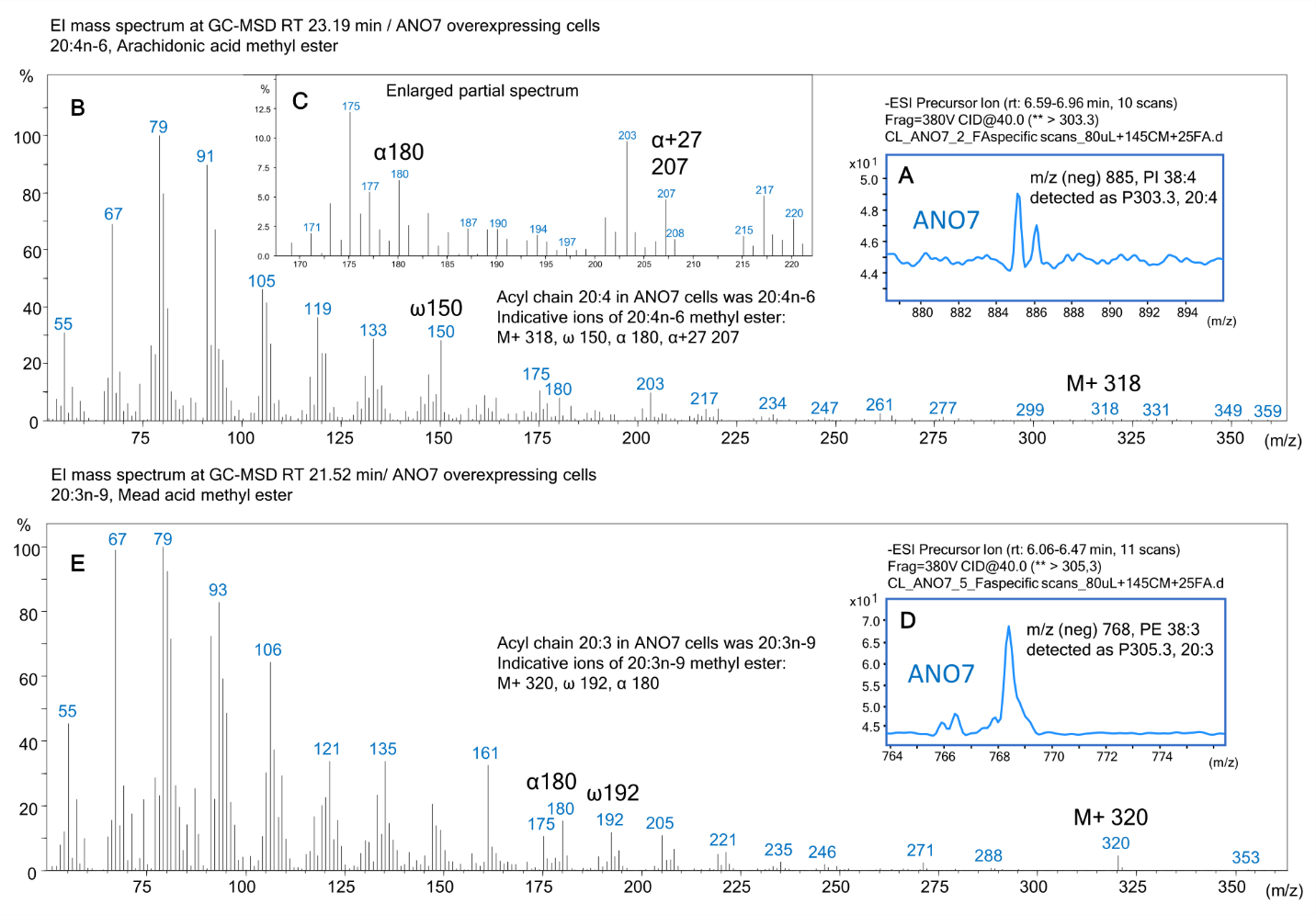


Figure S1. Identification of arachidonic acid 20:4n-6 and mead acid 20:3n9 present in several phospholipid species of ANO7 overexpressing cells. (A) ESI-MS/MS Precursor scan of m/z303.3 indicated that phosphatidylinositol PI38:4 (m/z885) contained a polyunsaturated acyl chain 20:4. (B) This 20:4 was shown by GC-MSD EI spectrum to be arachidonic acid 20:4n-6 based on indicative ions: M+ 318, ω 150, α 180 and α+27 207. (C) The fragments α 180 and α+27 207 are easier to recognize in the enlarged partial spectrum extracted from the top of the total ion chromatogram peak of the fatty acid. (D) ESI-MS/MS Precursor scan of m/z305.3 indicated that phosphatidylethanolamine PE38:3 (m/z768) contained a polyunsaturated acyl chain 20:3. (B) This 20:3 was shown by GC-MSD EI spectrum to be mead acid20:3n-9 based on indicative ions: M+320, ω 192 and α 180. M+ is the molecular ion, the ω fragment is from the cleaved bond towards the methyl end, and the α fragment towards the methyl ester end. Abbreviations of the ionization method: ESI = electrospray ionization, EI = electron impact. In panels (B), (C) and (E), the y axis % value represents ion intensity normalized to the tallest (base) peak.

Figure S2.
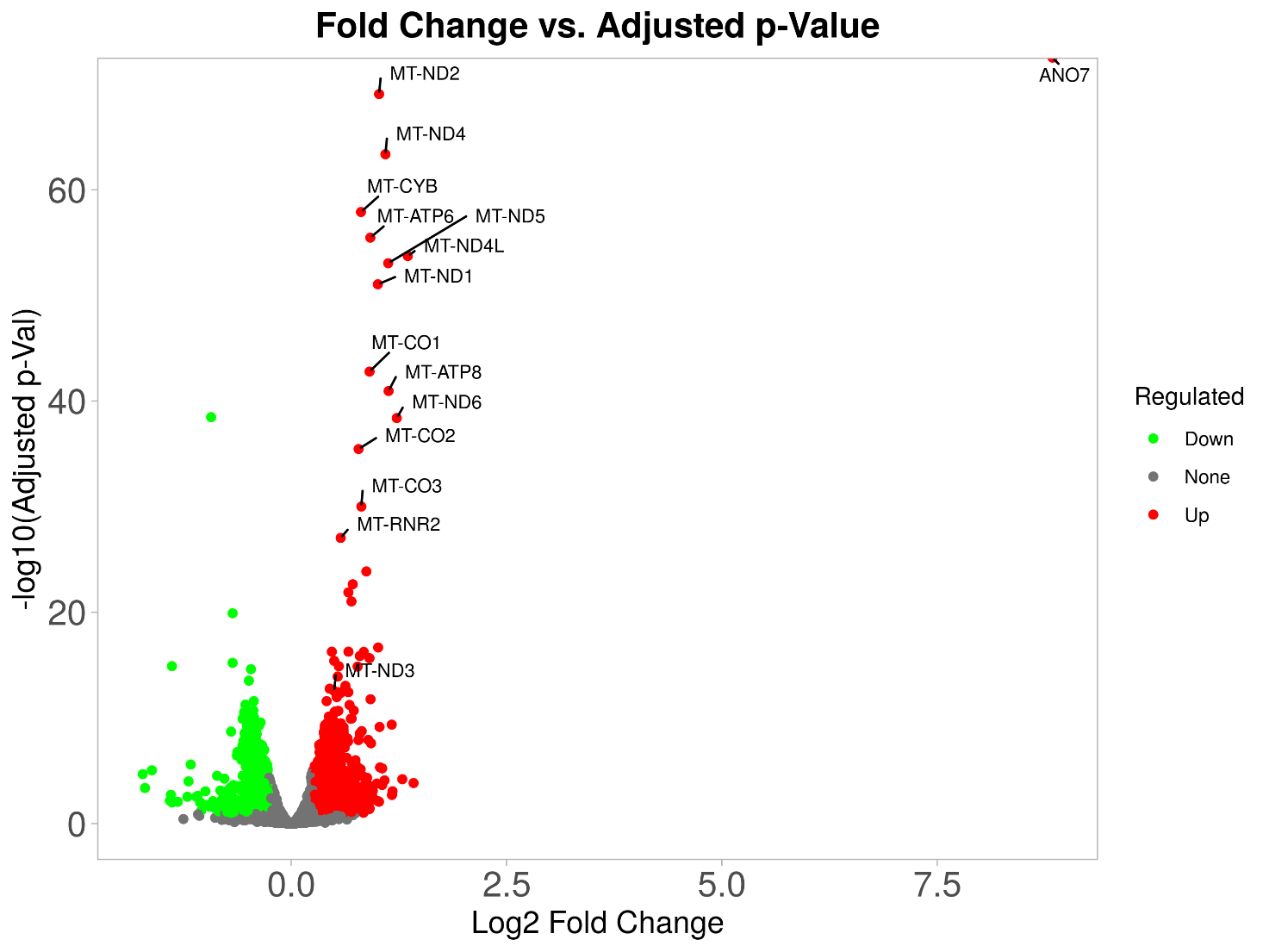
RNA-sequencing revealed that transcripts originating from the mitochondrial genome are among the most upregulated genes in RWPE1 cells expressing ANO7.


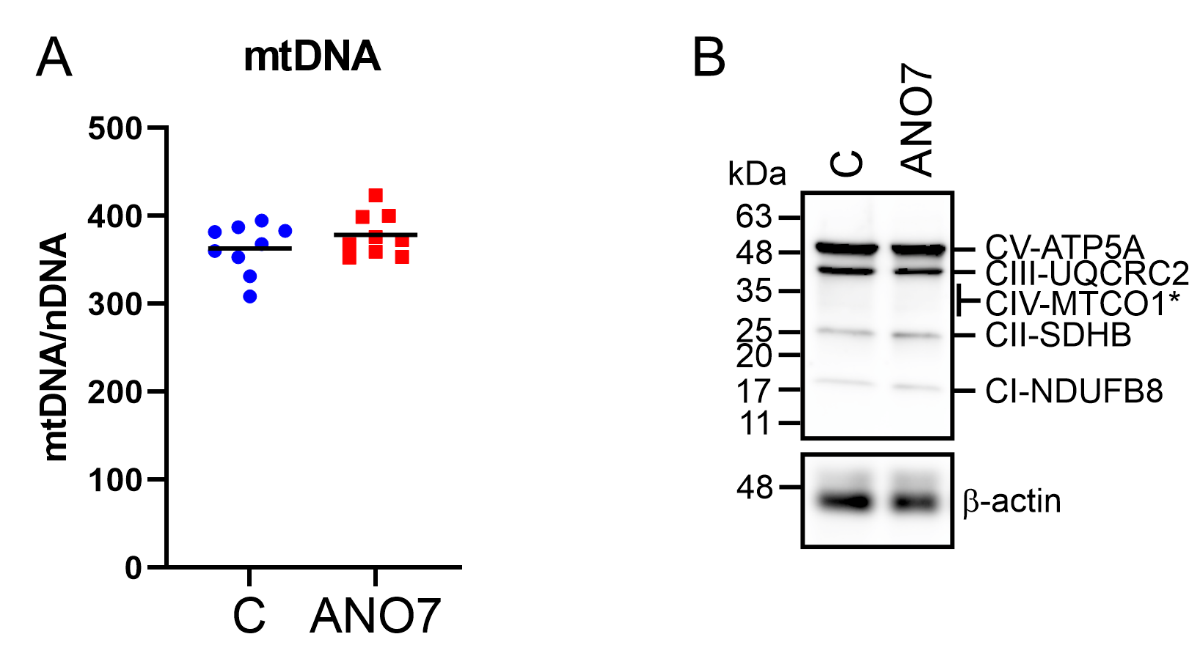


Figure S3. (A) ANO7 expression does not affect mtDNA levels in RWPE1 cells as measured with ddPCR. (B) No significant changes in the different mitochondrial respiratory complex markers could be detected between RWPE1 control and ANO7 cells (n=4). * the complex IV marker MTCO1, which is a highly hydrophobic protein, appeared as smear in our gel system.

| Geneset | SetSize | EnrichmentScore | NES | p-value | padj-value |
| --- | --- | --- | --- | --- | --- |
| HALLMARK_MYC_TARGETS_V1 | 199 | -0.786758873 | -4.429510874 | 0.002004008 | 0.003367003 |
| HALLMARK_E2F_TARGETS | 198 | -0.782636494 | -4.398289813 | 0.002 | 0.003367003 |
| HALLMARK_G2M_CHECKPOINT | 193 | -0.702305869 | -3.932000449 | 0.001996008 | 0.003367003 |
| HALLMARK_OXIDATIVE_PHOSPHORYLATION | 185 | -0.694328044 | -3.858879554 | 0.001996008 | 0.003367003 |
| HALLMARK_MTORC1_SIGNALING | 199 | -0.676710536 | -3.809930567 | 0.002004008 | 0.003367003 |
| HALLMARK_DNA_REPAIR | 147 | -0.692913096 | -3.712984927 | 0.001848429 | 0.003367003 |
| HALLMARK_MYC_TARGETS_V2 | 58 | -0.746610695 | -3.357477909 | 0.001424501 | 0.003367003 |
| HALLMARK_UNFOLDED_PROTEIN_RESPONSE | 112 | -0.630835519 | -3.247999813 | 0.001642036 | 0.003367003 |
| HALLMARK_P53_PATHWAY | 197 | -0.573998729 | -3.222250721 | 0.002012072 | 0.003367003 |
| HALLMARK_MITOTIC_SPINDLE | 198 | -0.547421677 | -3.076420798 | 0.002 | 0.003367003 |
| HALLMARK_PROTEIN_SECRETION | 95 | -0.596232069 | -2.969906374 | 0.001615509 | 0.003367003 |
| HALLMARK_GLYCOLYSIS | 198 | -0.497340504 | -2.79497275 | 0.002 | 0.003367003 |
| HALLMARK_REACTIVE_OXYGEN_SPECIES_PATHWAY | 48 | -0.594273023 | -2.553185904 | 0.001383126 | 0.003367003 |
| HALLMARK_UV_RESPONSE_UP | 157 | -0.463458018 | -2.523258234 | 0.001879699 | 0.003367003 |
| HALLMARK_HEME_METABOLISM | 197 | -0.438975332 | -2.464271278 | 0.002012072 | 0.003367003 |
| HALLMARK_PI3K_AKT_MTOR_SIGNALING | 105 | -0.475500162 | -2.426473519 | 0.001607717 | 0.003367003 |
| HALLMARK_CHOLESTEROL_HOMEOSTASIS | 74 | -0.503644735 | -2.369279319 | 0.00152439 | 0.003367003 |
| HALLMARK_ADIPOGENESIS | 195 | -0.405515095 | -2.266330457 | 0.002020202 | 0.003367003 |
| HALLMARK_TGF_BETA_SIGNALING | 54 | -0.499779418 | -2.194634747 | 0.001438849 | 0.003367003 |
| HALLMARK_INTERFERON_ALPHA_RESPONSE | 95 | -0.401870652 | -2.001767888 | 0.001615509 | 0.003367003 |
| HALLMARK_FATTY_ACID_METABOLISM | 157 | -0.361124783 | -1.966113535 | 0.001879699 | 0.003367003 |
| HALLMARK_APOPTOSIS | 161 | -0.354721896 | -1.943583169 | 0.001879699 | 0.003367003 |
| HALLMARK_TNFA_SIGNALING_VIA_NFKB | 199 | -0.33536109 | -1.888107838 | 0.002004008 | 0.003367003 |
| HALLMARK_HYPOXIA | 194 | -0.330080293 | -1.846024726 | 0.002008032 | 0.003367003 |
| HALLMARK_ESTROGEN_RESPONSE_LATE | 198 | -0.291607229 | -1.638785204 | 0.002 | 0.003367003 |
| HALLMARK_PEROXISOME | 104 | -0.32079322 | -1.636381898 | 0.001569859 | 0.003367003 |
| HALLMARK_SPERMATOGENESIS | 130 | -0.291064275 | -1.541545668 | 0.005338078 | 0.008087997 |
| HALLMARK_APICAL_JUNCTION | 198 | -0.255004241 | -1.433082362 | 0.006 | 0.008823529 |
| HALLMARK_XENOBIOTIC_METABOLISM | 200 | -0.230987156 | -1.306157636 | 0.0256917 | 0.035682916 |
| HALLMARK_BILE_ACID_METABOLISM | 112 | 0.294556551 | 1.371306793 | 0.037329505 | 0.049117769 |
| HALLMARK_COAGULATION | 138 | 0.288353633 | 1.389025199 | 0.023480663 | 0.033543804 |
| HALLMARK_IL6_JAK_STAT3_SIGNALING | 86 | 0.315999281 | 1.405031527 | 0.036929761 | 0.049117769 |
| HALLMARK_INFLAMMATORY_RESPONSE | 199 | 0.288702704 | 1.453820279 | 0.003992016 | 0.006237525 |
| HALLMARK_EPITHELIAL_MESENCHYMAL_TRANSITION | 198 | 0.294826088 | 1.483720164 | 0.002663116 | 0.004295348 |
| HALLMARK_KRAS_SIGNALING_DN | 194 | 0.346445941 | 1.740694858 | 0.000664894 | 0.003367003 |
| HALLMARK_MYOGENESIS | 200 | 0.348352323 | 1.753690347 | 0.000668449 | 0.003367003 |
| HALLMARK_KRAS_SIGNALING_UP | 198 | 0.363119546 | 1.827408817 | 0.000665779 | 0.003367003 |
| HALLMARK_ALLOGRAFT_REJECTION | 199 | 0.424144148 | 2.135862791 | 0.000665336 | 0.003367003 |

Table S1. ANO7 corGSEA results obtained from the Correlation AnalyzeR tool performed on normal prostate tissue samples in ARCHS4 database.
